# Two Novel Heat-Soluble Protein Families Abundantly Expressed in an Anhydrobiotic Tardigrade

**DOI:** 10.1371/journal.pone.0044209

**Published:** 2012-08-28

**Authors:** Ayami Yamaguchi, Sae Tanaka, Shiho Yamaguchi, Hirokazu Kuwahara, Chizuko Takamura, Shinobu Imajoh-Ohmi, Daiki D. Horikawa, Atsushi Toyoda, Toshiaki Katayama, Kazuharu Arakawa, Asao Fujiyama, Takeo Kubo, Takekazu Kunieda

**Affiliations:** 1 Department of Biological Sciences, Graduate School of Science, The University of Tokyo, Tokyo, Japan; 2 Institute of Medical Science, The University of Tokyo, Tokyo, Japan; 3 Center for Genetic Resource Information, National Institute of Genetics, Mishima, Shizuoka, Japan; 4 Human Genome Center, Institute of Medical Science, The University of Tokyo, Tokyo, Japan; 5 Institute for Advanced Biosciences, Keio University, Fujisawa, Kanagawa, Japan; 6 Principles of Informatics Research Division, National Institute of Informatics, Tokyo, Japan; Louisiana State University and A & M College, United States of America

## Abstract

Tardigrades are able to tolerate almost complete dehydration by reversibly switching to an ametabolic state. This ability is called anhydrobiosis. In the anhydrobiotic state, tardigrades can withstand various extreme environments including space, but their molecular basis remains largely unknown. Late embryogenesis abundant (LEA) proteins are heat-soluble proteins and can prevent protein-aggregation in dehydrated conditions in other anhydrobiotic organisms, but their relevance to tardigrade anhydrobiosis is not clarified. In this study, we focused on the heat-soluble property characteristic of LEA proteins and conducted heat-soluble proteomics using an anhydrobiotic tardigrade. Our heat-soluble proteomics identified five abundant heat-soluble proteins. All of them showed no sequence similarity with LEA proteins and formed two novel protein families with distinct subcellular localizations. We named them Cytoplasmic Abundant Heat Soluble (CAHS) and Secretory Abundant Heat Soluble (SAHS) protein families, according to their localization. Both protein families were conserved among tardigrades, but not found in other phyla. Although CAHS protein was intrinsically unstructured and SAHS protein was rich in β-structure in the hydrated condition, proteins in both families changed their conformation to an α-helical structure in water-deficient conditions as LEA proteins do. Two conserved repeats of 19-mer motifs in CAHS proteins were capable to form amphiphilic stripes in α-helices, suggesting their roles as molecular shield in water-deficient condition, though charge distribution pattern in α-helices were different between CAHS and LEA proteins. Tardigrades might have evolved novel protein families with a heat-soluble property and this study revealed a novel repertoire of major heat-soluble proteins in these anhydrobiotic animals.

## Introduction

Water is essential for life and most animals cannot survive without water. Some organisms, including tardigrades, however, are able to tolerate an almost complete loss of water by entering a metabolically inactive state, referred to as anhydrobioisis, and they can resume their activity upon rehydration [1], [2]. Dehydrated tardigrades showed extraordinary tolerance against various physical extremes including exposure to space [3]–[6], but the molecular basis of these tolerant abilities is totally unknown. The anhydrobiotic ability was observed in several species belonging to four animal phyla; arthropods, nematodes, rotifers and tardigrades. In anhydrobiotic arthropods and nematodes, trehalose has long been suggested to have an important role in desiccation tolerance because it accumulates in large amounts (∼15%–20% of body weight) upon desiccation [7]–[9]. In contrast, accumulation of trehalose was much less in tardigrades, varying from 0% to at most 2.9% (less than 1% in most species) [10]–[12], which suggests that tardigrades have other factors to tolerate dehydration. Another candidate molecule is the late embryogenesis abundant (LEA) protein family. LEA proteins were originally identified as abundant proteins in maturing plant seeds and their expression was significantly induced by desiccation in anhydrobiotic animals other than tardigrades [13]–[15]. The LEA proteins maintain their solubility even after heat-treatment, and are proposed to prevent protein-aggregation by interfering close association of damaged proteins as ‘molecular shield’ in a dehydrated condition [16]–[18]. Although the presence of LEA-like transcripts and protein were detected by expressed sequence tag (EST) analyses or proteomics of tardigrades [19]–[22], the induced expressions by desiccation and their biochemical property including heat-solubility have not been clarified and thus, their relevance to desiccation tolerance is obscure in tardigrades.

Here, to elucidate the molecular basis of tardigrade anhydrobiosis, we utilized the heat-soluble property characteristic of LEA proteins and searched for major heat-soluble proteins from an anhydrobiotic tardigrade, *Ramazzottius varieornatus*. We previously showed *R. varieornatus* have high tolerant ability against desiccation [23] and its genome sequences have been determined by our group. Thus this species is a suitable model for molecular analysis of tardigrade tolerant abilities. Our heat-soluble proteomics identified five abundant heat-soluble proteins forming two novel protein families with distinct subcellular localizations. No LEA proteins were detected. Both protein families were conserved among tardigrades, but not found in other phyla. Water-deficient conditions induced conformational changes of proteins in both families to an α-helices as LEA proteins. Two conserved repeats of 19-mer motifs in CAHS proteins were capable to form amphiphilic stripes in α-helices, suggesting their roles as molecular shield in water-deficient condition. Tardigrades might have evolved novel protein families different from LEA proteins and this study revealed novel repertoire of major heat-soluble proteins in anhydrobiotic animals.

## Materials and Methods

### Animals

We used a strain, YOKOZUNA-1, which was established from a single individual of *R. varieornatus*. The same strain was used for our ongoing genome sequencing project of *R. varieornatus*. Tardigrades were reared on agar plates by feeding algae, *Chlorella vulgaris* purchased from Chlorella Industry Co., Ltd. (Japan) as described previously [23].

### Heat-soluble proteomics

Tardigrades were starved for 2 days to eliminate food contamination, and then 500 individuals were homogenized well manually using disposable plastic pestle (Kontes) in 40 µl ice-cold PBS (137 mM NaCl, 2.7 mM KCl, 10 mM Na_2_HPO_4_, 1.76 mM KH_2_PO_4_, pH = 7.4) with protease inhibitors, Complete (Roche). ‘Pre-heat’ soluble protein fraction was collected after centrifugation at 12,000 rpm for 20 min and heated at 92°C for 15 min. The heat-soluble fraction was separated as supernatants by centrifugation at 12,000 rpm for 20 min. Proteins were analyzed by SDS-PAGE and detected with Flamingo Fluorescent Gel Stain (Bio-Rad).

### Protein identification

Heat-soluble protein bands were excised and digested in trypsin. Tryptic peptides were separated by nanoflow LC and analyzed using a MALDI-TOF/TOF 4800 proteomics analyzer (Applied Biosystems). Corresponding peptide sequences were retrieved from 6 frame translations of our in-house draft genome assembly of YOKOZUNA-1 strain using MASCOT software (Matrix Science). The draft genome assembly used for this study covered more than 90% of estimated genome size and will be published elsewhere. Rapid amplification of cDNA ends (RACE) was performed to determine full-length cDNA sequences using the First-Choice RLM-RACE Kit (Invitrogen). The nucleotide sequences reported in this study have been submitted to DDBJ with accession numbers AB650497-AB650501.

### Sequence analyses

Subcellular localizations and signal peptides were predicted by TargetP and SignalP programs, respectively [24], [25]. Sequences of other tardigrade homologs were retrieved from NCBI databases of EST and transcriptome shotgun assembly (TSA). EST sequences for CAHS and SAHS gene homologs were assembled using Codon Code Aligner (Codon Code Corp.). Accession numbers used in these assemblies were listed in Tables S1, S2, and S3. ESTs coding actin or elongation factor 1 alpha (EF1α) were retrieved by querying the sequences of *Drosophila melanogaster*; NP_727048 for actin and AAF58608 for EF1α. Accession numbers for ESTs of actin and EF1α were listed in Table S4. Multiple alignments and sequence Logo analyses were performed using Geneious software (Biomatters Ltd.).

### Heat-solubility assay of recombinant proteins

Recombinant proteins were expressed in *Escherichia coli* BL21(DE3) strain using pET system (Novagen) in fusion with a His-tag at the N-terminus. Signal peptides were excluded for the secretory proteins. After bacterial cells were lysed in PBS with protease inhibitors by sonication, the soluble fraction was collected and served as pre-heat sample. After heat-treatment, heat-soluble and heat-insoluble fractions were separated by centrifugation and analyzed by SDS-PAGE.

### Subcellular localization

To utilize the ease in visualization of subcellular components and high transfection efficiency, we used adherent mammalian cells. For expression of green fluorescent protein (GFP)-fused proteins, coding sequences were inserted into pAcGFP1-N1 (Clontech). Human embryonic kidney 293T cells were transfected with the construct using X-tremeGENE 9 reagent (Roche). After 24 to 48 hours incubation in Dulbecco's Modified Eagle Medium with 10% fetal bovine serum, cells were stained with Hoechst 33342 (Lonza) and MitoTracker Red CMXRos (Lonza), to visualize nuclear DNA and mitochondria respectively. Fluorescent signals were observed by confocal microscopy LSM710 (Carl Zeiss).

### Immunoblot analysis

Transfected cells and culture medium were separated by centrifugation and subjected to SDS-PAGE. GFP-fusion protein was reacted with anti-GFP rabbit polyclonal antibody (Invitrogen) and detected by chemiluminescent reagent LumiGLO (Cell Signaling Tech.).

### Circular Dichroism (CD) spectroscopy

His-tagged protein was expressed using a pET system (Novagen) and purified with Ni-NTA agarose (Invitrogen). After dialysis to remove the imidazole, the CD spectra were measured using an AVIV 202SF CD spectrometer.

## Results

### Novel abundant heat-soluble proteins in tardigrades

**Figure 1:**
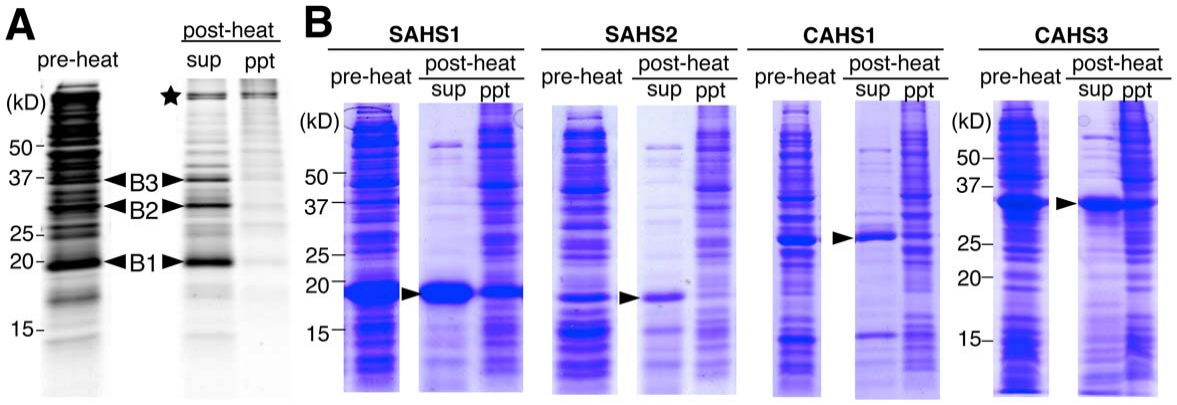
Heat soluble proteomics. (A) The soluble fraction of tardigrade lysate (‘pre-heat’) was heated and separated to heat-soluble fraction (sup) and insoluble precipitate (ppt) by centrifugation. Arrows indicate the heat-soluble bands detected selectively in the heat-soluble fraction, named B1, B2, and B3. Star indicates the partially heat-soluble bands detected in both the supernatant and precipitate after heat treatment. (B) Heat solubility of bacterial lysate expressing SAHS and CAHS proteins. Arrows indicate each SAHS or CAHS protein.

Because LEA proteins maintain their solubility after heat treatment and are believed to have an important role in anhydrobiosis, we searched for major heat-soluble proteins from an anhydrobiotic tardigrade, *R. varieornatus*. The soluble protein fraction of the tardigrade lysate served as the pre-heat sample and was heated at 92°C for 15 min. Heat-soluble proteins were recovered as supernatant after centrifugation and analyzed by gel electrophoresis. As shown in Fig. 1A, three major bands, termed B1 to B3, were detected selectively in the heat-soluble fraction, though most other proteins disappeared after heat-treatment even in the insoluble fraction, possibly due to the severe aggregation caused by heat, which prevented separation of the proteins by gel electrophoresis. These heat-soluble bands were detected with similar intensity in pre-heat condition, suggesting that the proteins in these bands were fairly heat-soluble.

**Figure 2:**
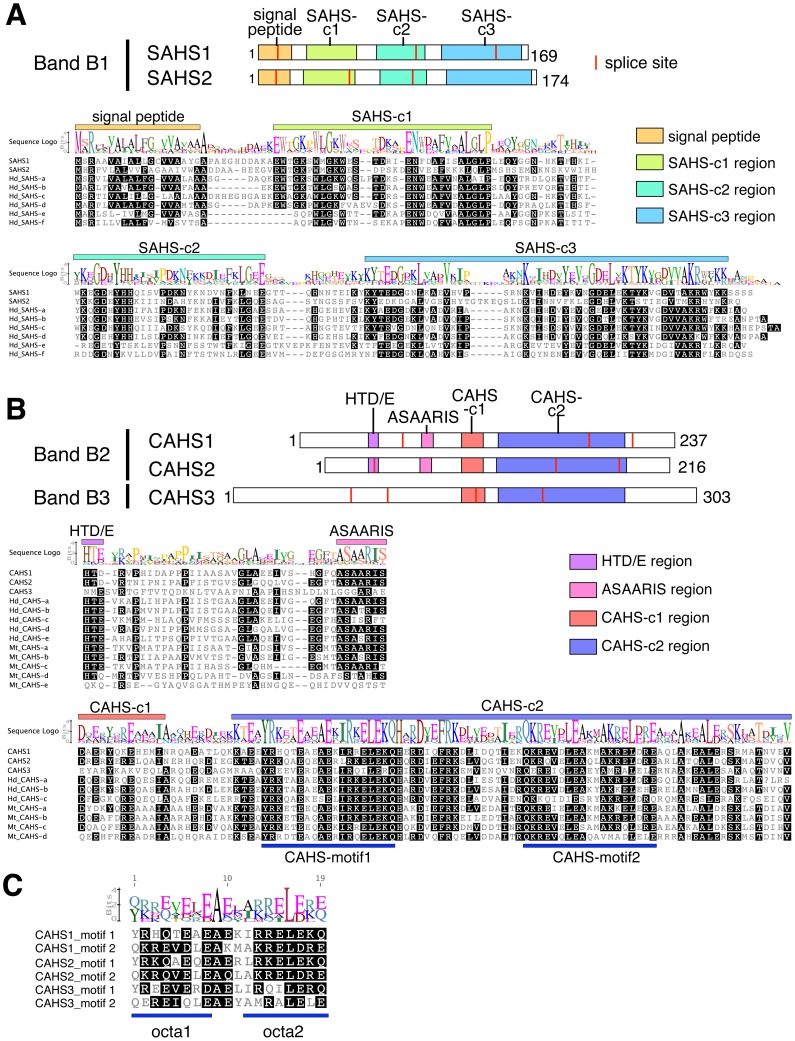
Gene structures and conserved motifs of SAHS and CAHS proteins among tardigrades. (A) Schematic representation of SAHS1 and SAHS2 proteins. Splice sites were not conserved between them. Multiple alignments of SAHS1, SAHS2 and 6 SAHS homologs of *H. dujardini* (Hd_SAHSs) revealed 3 conserved regions. Hydrophobic secretory signals were found at the N-terminus in all SAHS homologs. (B) Schematic representations of CAHS proteins and multiple alignments among CAHS homologs; 3 proteins from *R. varieornatus* and 5 homologs each from *H. dujardini* (Hd_CAHSs) and *M. tardigradum* (Mt_CAHSs). There are 4 conserved regions. Two repeats of 19 mer CAHS-motifs were conserved in the CAHS-c2 region. (C) Multiple alignments of 6 CAHS-motif sequences from *R. varieornatus*. Two octapeptides (octa1, octa2) were repeated with a tripeptide linker.

Mass spectrometry of these bands was separately analyzed and corresponding peptide sequences were retrieved from the draft genome database. Based on the retrieved sequences, we applied the RACE method and determined full length cDNA sequences for a total of five proteins. The estimated molecular weight of each protein coincided well with the mobility on the gel, suggesting the lack of post-translational modifications (Table S5). Two proteins identified from band B1 were similar to each other (37% identity). Although no Pfam motifs were found, a BLASP search against a protein database revealed low similarity with mammalian fatty acid binding proteins (FABPs) (Table S5). In contrast to mammalian FABPs, which are cytosolic and contain lipocalin motifs [26], the newly identified proteins contained no motif and instead contained a secretory signal-peptide at N-terminus (Fig. 2A). Thus, these proteins formed a FABP-related, but distinct secretory protein family. We designated this protein family the Secretory Abundant Heat Soluble (SAHS) protein family. Three other heat-soluble proteins identified from bands B2 and B3 also shared several conserved sequences and formed another protein family without secretory signals (Fig. 2B). These proteins showed no similarity with known sequences in BLASTP search and no known motifs were found in Pfam database, suggesting that they formed a novel protein family. We named this group the Cytoplasmic Abundant Heat Soluble (CAHS) protein family. Notably, no LEA proteins were detected in mass spectrometry of the predominant heat-soluble bands, suggesting that LEA proteins are not major heat-soluble proteins in the tardigrades.

Because our initial heat-soluble proteomics was performed using the lysate of tardigrades, certain internal solutes of tardigrades such as sugars might have contributed to the heat-solubility of the identified proteins. To examine this possibility, we expressed recombinant proteins in *E. coli* for two members of each SAHS/CAHS protein family as representatives and subjected the bacterial lysate to a heat solubility assay. After heat treatment, bacterially expressed SAHS and CAHS proteins were recovered mainly in the heat-soluble fractions (Fig. 1B). These results suggest that the heat-solubility of SAHS and CAHS proteins does not require the presence of other solutes or post-translational modifications.

### SAHS and CAHS are conserved among tardigrades

Comparison of cDNA sequences and genome sequences revealed the genomic structure of SAHS and CAHS proteins (Fig. S1). In both protein families, all of their splice sites were not conserved among family members, suggesting that most introns have been inserted after the divergence of paralogous members during evolution (Fig. 2A, 2B, S1). A TBLASTN search against the EST or TSA databases in NCBI retrieved significantly similar sequences from two other tardigrade species, *Hypsibius dujardini* and *Milnesium tardigradum*. The assembly of corresponding EST sequences revealed 6 SAHS and 5 CAHS genes in *H. dujardini*. In this species, the total number of ESTs coding SAHS proteins was 100 and was much higher than those for actin and EF1α, which were 15 and 64 respectively (Table S4). The number of ESTs for CAHS was 23 and also higher than that of actin. These results suggested that SAHS and CAHS genes were abundantly expressed in *H. dujardini* as well. Although 5 CAHS genes were found in the TSA database of *M. tardigradum*, no SAHS gene was found in the transcriptomic database of this species at present. Phylogenetically, *R. varieornatus* has closer relationship with *H. dujardini* than *M. tardigradum* and thus, the abundant expression of SAHS genes might be a common characteristic in the clade containing *R. varieornatus* and *H. dujardini*. Comparison of these homologous genes revealed three and four conserved regions for SAHS and CAHS proteins, respectively (Fig. 2A, 2B). Detailed inspection of conserved regions revealed two repeats of 19-mer peptides in the CAHS-c2 region (Fig. 2B). We designated these as CAHS-motifs. Each CAHS motif comprised two octapeptides connected by a tripeptide (Fig. 2C). The consensus sequence of the octapeptide was ΦψψΩΦΩ(Φψ)ΩΦ, hydrophobic residue; ψ, basic residue; Ω, acidic and amide residue), which was distinct from the consensus 11-mer motif of LEA proteins, ΦΦΩXΦψΩψΦXΩ [27], [28]

### Distinct subcellular localization of CAHS and SAHS

**Figure 3:**
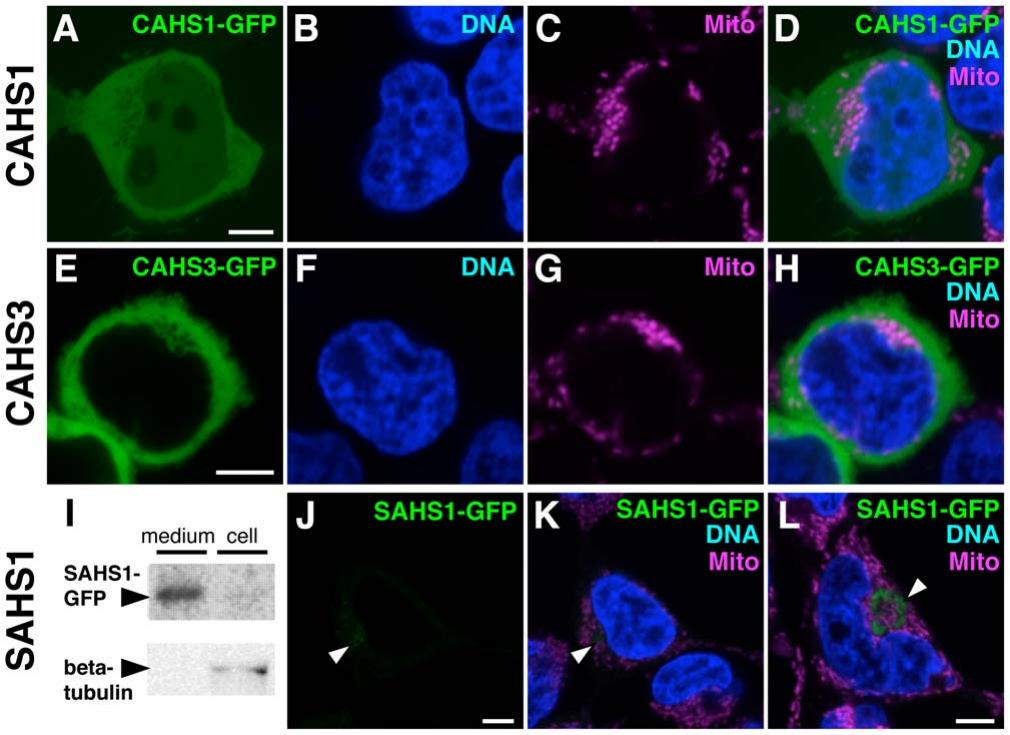
Distinct subcellular localization of CAHS and SAHS proteins. Pseudo-color fluorescent images of 293T cells expressing AcGFP fusion proteins of CAHS1 (A–D), CAHS3 (E–H) and SAHS1 (J–L). (A, E, J) AcGFP fusion proteins. (B, F) DNA. (C, G) mitochondria. (D, H, K, L) merged images. Bars indicate 5 µm. (I) Immunoblot analyses using anti-GFP and anti-β-tubulin antibody to examine whether SAHS1-GFP was secreted into the culture medium (medium) or retained inside the cells (cell).

Eukaryotic cells contain membrane-delineated compartments. Computer prediction of subcellular localization using TargetP program suggested that five proteins were targeted to three different locations; SAHS1 and SAHS2 are secretory; CAHS1 and CAHS2 are cytoplasmic; and CAHS3 is mitochondrial (Table S5). The prediction score for CAHS3 was low and its predicted mitochondrial localization was uncertain. To elucidate subcellular localizations of CAHS and SAHS proteins experimentally, we chose SAHS1, CAHS1 and CAHS3 as representative members of three differently predicted locations, and expressed their GFP-fusion proteins in cultured cells. CAHS1-GFP was distributed mostly in the cytoplasm and weakly in the nucleus (Fig. 3A–D), whereas CAHS3-GFP localized only in the cytoplasm and no signals were observed in the nucleus or mitochondria (Fig. 3E–H). Thus, CAHS proteins localize mainly in the cytoplasm and certain members can also distribute in the nucleus. In contrast, SAHS1-GFP fusion proteins were detected in the culture medium rather than the cell bodies by immunoblot analysis (Fig. 3I), suggesting that SAHS1 is a secretory protein. Fluorescent signals for SAHS1-GFP were hardly detected, but in a small population of cells, weak intracellular signals were observed (Fig. 3J–L), possibly due to proteins remaining in secretory pathway such as the endoplasmic reticulum or Golgi apparatus. SAHS might contribute to protect these secretory organelles.

### α-Helices induced by water-deficient conditions

**Figure 4:**
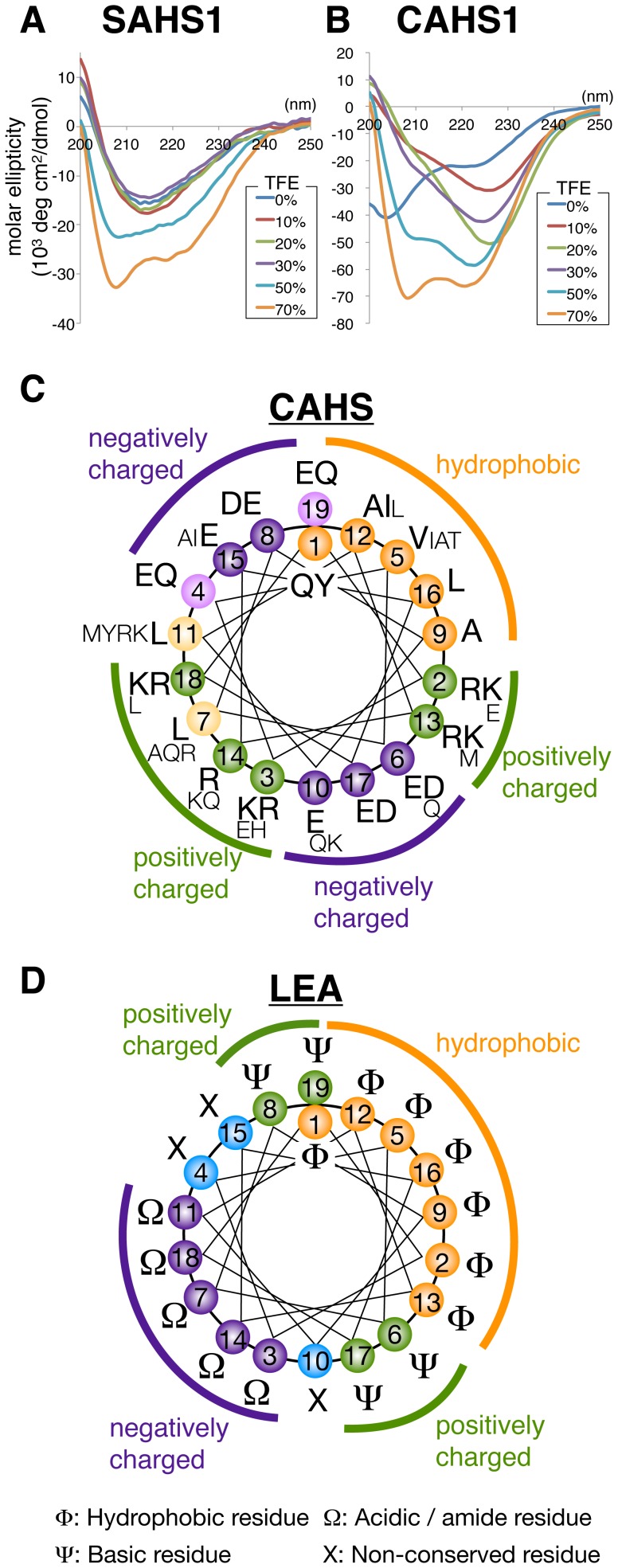
Dynamic change of secondary structures of SAHS and CAHS proteins. (A, B) CD spectra of SAHS1 and CAHS1 proteins in the absence or presence of various concentrations of the desolvating agent TFE, mimicking a water deficient condition. (C) Formation of 5 striped amphiphilic α-helices by CAHS repeats. The numbers show the positions in the repeat. Based on multiple alignments of 6 CAHS repeats, major amino acids at each position are shown in larger size and minor ones in smaller size. (D) Formation of 4 striped α-helices of conserved LEA motifs. Φ, aliphatic residue; ψ, basic residue; Ω, acidic or amide residue; X, non-conserved residue.

As all SAHS and CAHS proteins were predicted to be intrinsically unstructured similar to LEA proteins by the FoldIndex program [29] (Fig. S2), we examined their secondary structures using CD spectroscopy. In hydrated conditions, SAHS1 had a minimum at 215 nm, which is the typical CD spectrum of β-structures (Fig. 4A). FABP is rich in β-structures [30]. Thus, SAHS1 and FABP probably share similar secondary structures rather than the predicted unstructured conformation. In contrast, the CD spectrum of CAHS1 had a single deep minimum around 200 nm, similar to that of hydrated LEA proteins [31], suggesting that these proteins are unstructured in hydrated conditions (Fig. 4B). When water-deficient conditions were mimicked by increasing the desolvating agent of trifluoroethanol (TFE), the spectra of both SAHS1 and CAHS1 dynamically changed to the typical spectra of α-structures with minima at 206 nm and 221 nm (Fig. 4A, 4B) [31]. In contrast to SAHS1 which had robust β-structure and more than 50% TFE was required for the transition to α-helices, the CD spectrum of CAHS1 was drastically affected by the addition of only 10% of TFE, suggesting that CAHS1 is more sensitive to water availability. When amino acids of the 19-mer CAHS motif are aligned to α-helical structures, they are expected to form amphiphilic helices, with one hydrophobic stripe and a wider hydrophilic stripe, which can be further divided to four differently charged regions (Fig. 4C). The typical LEA motif is also expected to form amphiphilic helices, but its hydrophilic stripe is divided into three regions rather than four, and the charge distribution patterns were different between CAHS and LEA motifs (Fig. 4C, 4D).

## Discussion

In this study, we identified five novel abundant heat-soluble proteins forming two novel protein families from an anhydrobiotic tardigrade, *R. varieornatus*. Although there are several genes containing LEA-motifs in our draft genome database, no LEA proteins were detected by mass spectroscopy analysis of major heat-soluble bands. These results suggested that tardigrade LEA proteins are not heat-soluble or their expression level is much lower than those of CAHS or SAHS proteins. In any cases, this study established CAHS and SAHS proteins as major heat-soluble proteins in tardigrades. Proteins from both families become α-helical structure upon water deficient conditions. In particular, CAHS motifs form amphiphilic α-helices as LEA proteins do. These results suggested that in the tardigrade, the newly identified SAHS and CAHS proteins could support desiccation tolerance as abundant proteins in a similar manner to LEA proteins.

According to subcellular localization, CAHS protein may contribute to protection of cytoplasm and nucleus, while SAHS protein may protect extracellular components. No accumulation of SAHS1-GFP was observed around the plasma membranes, suggesting that SAHS1 has low affinity to membrane and membrane protection is not the major role of SAHS proteins. Our results did not exclude the possibility that SAHS proteins accumulate in a dehydration dependent manner and protect the membranes. None of CAHS and SAHS proteins localized in mitochondria, implying that mitochondria might be protected by other molecular species.

Reflecting the differences in primary sequences, the α-helices of CAHS motifs had one extra negatively-charged region compared to those of LEA motifs. As a result, each stripe became narrow and thus may prevent too strong hydrophobic or electro-static interaction with other molecules, possibly making CAHS proteins better stuffing molecules. Comparative proteomics using two-dimensional electrophoresis between hydrated and dehydrated tardigrades, revealed no significant reproducible changes (AY, unpublished data), raising the possibility that tardigrades constitutively expressed the factors necessary for anhydrobiosis. Weak interactive ability of CAHS motif might be appropriate for constitutive abundant expression.

Recent search for thermostable proteins from monogonont rotifer, *Brachionus manjavacas*, identified a LEA protein and vitellogenin as major heat soluble proteins in diapausing eggs of the rotifer, but no CAHS/SAHS proteins were found in the search [32]. CAHS and SAHS proteins were conserved among tardigrades, but were not found in other phyla, suggesting that tardigrades might have evolved novel heat-soluble proteins different from LEA proteins as major heat-soluble proteins. This study revealed a novel repertoire of major heat-soluble proteins in anhydrobiotic animals

## Supporting Information

Figure S1 **Genomic structures of SAHS and CAHS proteins.** (A) Both SAHS1 and SAHS2 contained four exons. Colored boxes and red lines indicated conserved regions and splice sites, respectively. (B) CAHS proteins are composed of four or five exons and their splice sites were not conserved among them.
(PDF)

Figure S2 **Predicted intrinsically unstructured regions of SAHS and CAHS proteins.** Y axis is foldIndex based on the net charges and residue hydrophobicity of the given sequence. The values beyond zero (green areas) show the tendency of a given amino acid for being ordered and the values below zero (red areas) show the tendency of a residue for being intrinsically unstructured.
(PDF)

Table S1 **Assembly of SAHS family members of** ***Hypsibius dujardini*** **.**
(PDF)

Table S2 **Assembly of CAHS family members of** ***Hypsibius dujardini*** **.**
(PDF)

Table S3 **Summary of CAHS family members of** ***Milnesium tardigradum*** **.**
(PDF)

Table S4 **Summary of ESTs coding housekeeping genes of** ***Hypsibius dujardini*** **.**
(PDF)

Table S5 **Summary of newly identified heat-soluble proteins from** ***Ramazzottius varieornatus*** **.**
(PDF)
