# Supplementary figures and images for: Two Novel Heat-Soluble Protein Families Abundantly Expressed in an Anhydrobiotic Tardigrade
Source: PLoS One. 2012 Aug 28;7(8):e44209. doi: 10.1371/journal.pone.0044209 (PMC3429414; doi:10.1371/journal.pone.0044209)

**A**

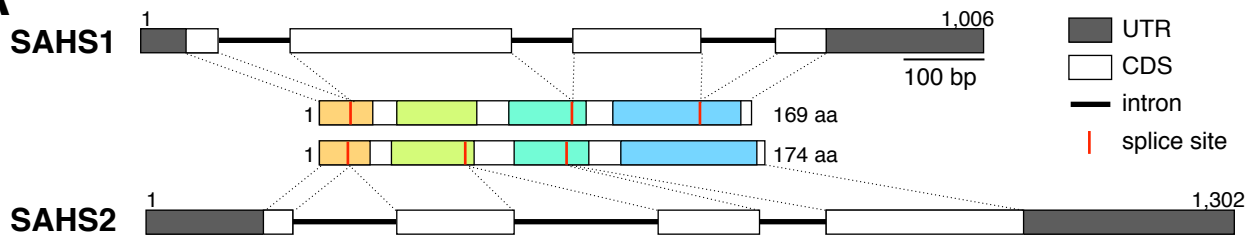

**B**

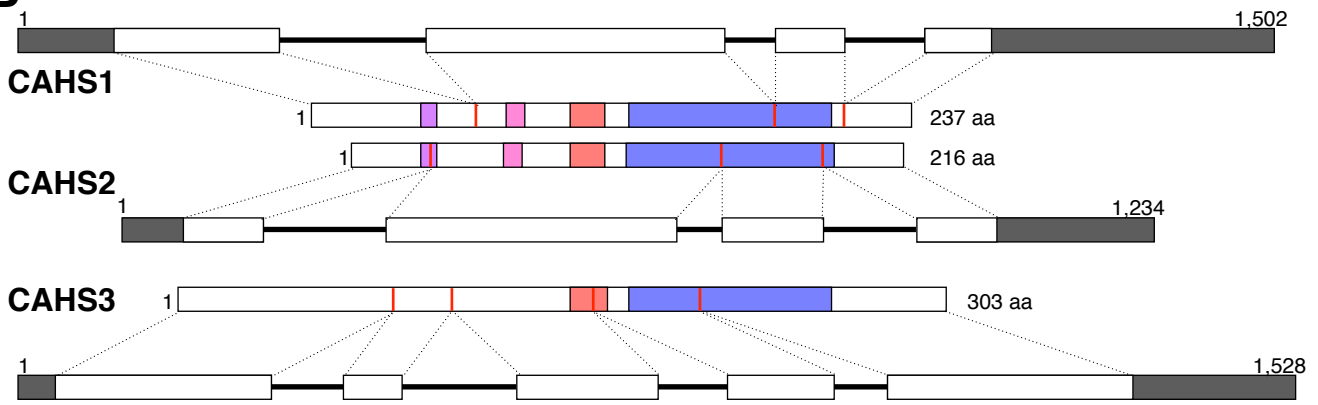

Supplement: Figure S1 — Genomic structures of SAHS and CAHS proteins. (A) Both SAHS1 and SAHS2 contained four exons. Colored boxes and red lines indicated conserved regions and splice sites, respectively. (B) CAHS proteins are composed of four or five exons and their splice sites were not conserved among them. (PDF) [file pone.0044209.s001.pdf]

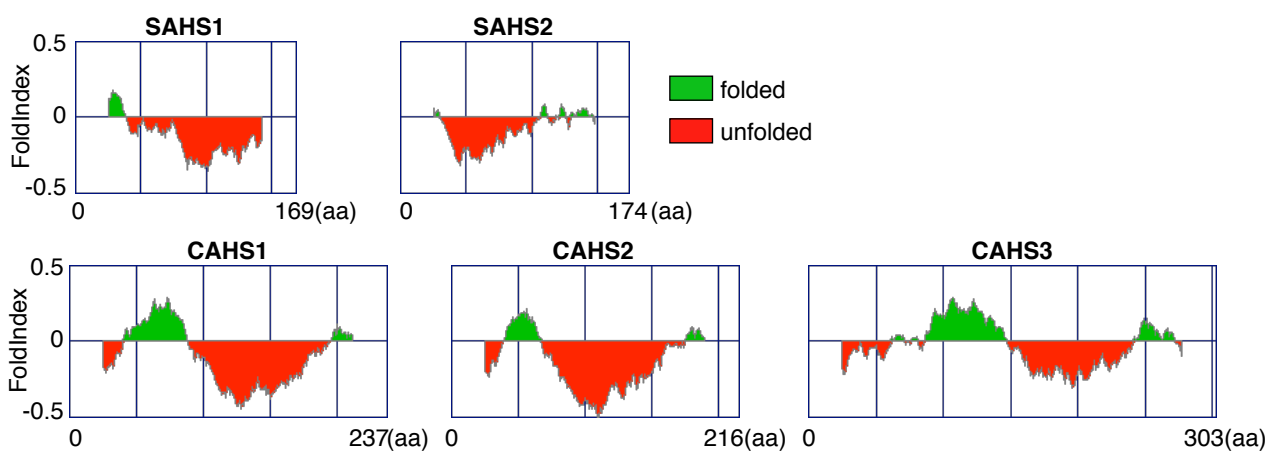

Supplement: Figure S2 — Predicted intrinsically unstructured regions of SAHS and CAHS proteins. Y axis is foldIndex based on the net charges and residue hydrophobicity of the given sequence. The values beyond zero (green areas) show the tendency of a given amino acid for being ordered and the values below zero (red areas) show the tendency of a residue for being intrinsically unstructured. (PDF) [file pone.0044209.s002.pdf]
