# Supplementary material for: Two Novel Heat-Soluble Protein Families Abundantly Expressed in an Anhydrobiotic Tardigrade
Source: PLoS One. 2012 Aug 28;7(8):e44209. doi: 10.1371/journal.pone.0044209 (PMC3429414; doi:10.1371/journal.pone.0044209)
Supplement: Table S1 — Assembly of SAHS family members of Hypsibius dujardini . (PDF) [file pone.0044209.s003.pdf]

Table S1.

Assembly of SAHS family members of *Hypsibius dujardini*

Contig_ID[a]	# of ESTs	Length (bp)	EST_IDs (Accession numbers)
Hd_SAHS-a	5	701	CO508084 CO508703 CO741517 CO741104 CO741989
Hd_SAHS-b	9	781	CO501891 CO508694 CO507967 CO741467 CK326538 CD449696 CK325834 CD449644 CO741700
Hd_SAHS-c	29	826	CO508015 CO508040 CO508391 CO508530 CO508014 CO508283 CO741565 CK326918 CK325830 CK326603 CK326878 CK326912 CO741870 CO741911 CO741193 CO741420 CO742019 CK326559 CF544461 CF076053 CF544672 CF075985 CD449738 CF076037 CF075909 CD449850 CD449553 CD449222 CK326833
Hd_SAHS-d	53	895	CO508087 CO508473 CO508593 CO508644 CO508656 CO508415 CO508062 CO507847 CO508506 CO741221 CO507885 CO508096 CO508336 CO508711 CO508476 CD449757 CF544675 CK326290 CK326297 CK326462 CK326527 CO741165 CO741725 CO741923 CO741978 CK326407 CK326524 CF544448 CF544706 CK325851 CD449073 CD449569 CD449817 CF075667 CF544557 CF544600 CF544641 CF544730 CK326028 CF075911 CF544507 CF544232 CF075706 CK325811 CF544386 CD449593 CF075958 CD449072 CK325959 CD449634 CO508179 CF544267 CK325779
Hd_SAHS-e	3	804	CO507834 CK326536 CF544671
Hd_SAHS-f	1	623	CO508579

[a]Totally 100 EST were retrieved by TBLASTN search of SAHS1 and SAHS2 protein sequences from NCBI EST database. All ESTs belong to *Hypsibius dujardini*. All EST sequences were assembled to five contigs and one singlet using Codon Code Aligner (CodonCode Corp.). All assemblies contain whole protein-coding regions.
