# Supplementary material for: Two Novel Heat-Soluble Protein Families Abundantly Expressed in an Anhydrobiotic Tardigrade
Source: PLoS One. 2012 Aug 28;7(8):e44209. doi: 10.1371/journal.pone.0044209 (PMC3429414; doi:10.1371/journal.pone.0044209)
Supplement: Table S2 — Assembly of CAHS family members of Hypsibius dujardini . (PDF) [file pone.0044209.s004.pdf]

Table S2.

Assembly of CAHS family members of *Hypsibius dujardini*

Contig_ID[a]	# of ESTs	Length (bp)	EST_IDs (Accession numbers)
Hd_CAHS-a	7	821	CF075767 CF544712 CO508419 CF544402 CK326497 CK326179 CK326856
Hd_CAHS-b	9	825	CO501870 CO508382 CO508483 CK326122 CK326586 CK326467 CK326537 CK326242 CK326124
Hd_CAHS-c	3	744	CF076089 CK325870 CF544452
Hd_CAHS-d[b]	2	683	CO508126 CO741378
Hd_CAHS-e[b]	2	696	CO508705 CO508488

[a]Totally 23 EST were retrieved by TBLASTN search of CAHS1, CAHS2 and CAHS3 protein sequences from NCBI EST database. All EST belong to *Hypsibius dujardini*. All EST sequences were assembled to five contigs using Codon Code Aligner (CodonCode Corp.). All assemblies contain N-terminus but lack C-terminus of protein-coding regions.

[b]Two assemblies were too short to cover full CAHS-c2 motif.
