# Supplementary material for: Two Novel Heat-Soluble Protein Families Abundantly Expressed in an Anhydrobiotic Tardigrade
Source: PLoS One. 2012 Aug 28;7(8):e44209. doi: 10.1371/journal.pone.0044209 (PMC3429414; doi:10.1371/journal.pone.0044209)
Supplement: Table S3 — Summary of CAHS family members of Milnesium tardigradum . (PDF) [file pone.0044209.s005.pdf]

Table S3.

Summary of CAHS family members of *Milnesium tardigradum*

Contig_ID[a]	cDNA (bp)	Protein (aa)	Accession numbers (TSA)
Mt_CAHS-a	1,392	259	EZ761416
Mt_CAHS-b	1,230	254	EZ763024
Mt_CAHS-c[b]	797	265	EZ760591
Mt_CAHS-d[b]	1,154	339	EZ758904
Mt_CAHS-e[c]	876	202	EZ761505

[a]Five CAHS family members of *Milnesium tardigradum* were retrieved by TBLASTN search of CAHS1, CAHS2 and CAHS3 protein sequences from NCBI TSA database.

[b]Two assemblies contain N-terminus but lack C-terminus of protein-coding regions.

[c]One assembly was too short to cover full CAHS-c2 motif.
