# Supplementary material for: Two Novel Heat-Soluble Protein Families Abundantly Expressed in an Anhydrobiotic Tardigrade
Source: PLoS One. 2012 Aug 28;7(8):e44209. doi: 10.1371/journal.pone.0044209 (PMC3429414; doi:10.1371/journal.pone.0044209)
Supplement: Table S4 — Summary of ESTs coding housekeeping genes of Hypsibius dujardini . (PDF) [file pone.0044209.s006.pdf]

Table S4.

Summary of ESTs coding housekeeping genes of *Hypsibius dujardini*

gene	# of ESTs	EST_IDs (Accession numbers)
Actin	15	CF544450 CK326228 CF075905 CF544576 CF544591 CF075811 CD449699 CF544173 CK326743 CF544544 CF075895 CD449282 CO741197 CK326842 CO741783
Elongation factor-1 alpha	64	CF544610 CF075937 CF075972 CD449442 CF544628 CD449420 CD449706 CK325998 CF544532 CK325995 CK325900 CF544224 CO507975 CK326039 CF075952 CK325929 CK325794 CD449183 CK326033 CO508373 CK325787 CF544177 CF076099 CF075722 CD449458 CF544664 CF544155 CF544716 CK325935 CD449219 CK326072 CO501862 CF544391 CD449878 CF544653 CF075900 CD449515 CD449831 CD449650 CF075731 CD449208 CK326134 CF075904 CK325792 CD449310 CD449927 CD449484 CF075843 CK325841 CD449945 CO741996 CO741174 CO741598 CO741937 CD449162 CD449475 CF075792 CF544459 CK326057 CK326693 CO501895 CD449521 CK326042 CO741339
