# Supplementary material for: Two Novel Heat-Soluble Protein Families Abundantly Expressed in an Anhydrobiotic Tardigrade
Source: PLoS One. 2012 Aug 28;7(8):e44209. doi: 10.1371/journal.pone.0044209 (PMC3429414; doi:10.1371/journal.pone.0044209)
Supplement: Table S5 — Summary of newly identified heat-soluble proteins from Ramazzottius varieornatus . (PDF) [file pone.0044209.s007.pdf]

Table S5.

Summary of newly identified heat-soluble proteins from *Ramazzottius varieornatus*

Band	Protein	MW (Da)	cDNA (bp)	Protein (aa)	Coverage (%)	pI	BLASTP (e-value)	Localization[c] (score)
B1	SAHS1	19,130<br>17,312[a]	752	169	50	7.0	Pa_FABP[b] (2e-06)	Secretory (0.910)
	SAHS2	19,878<br>17,825[a]	918	174	51	7.5	Bb_FABP[b] (7e-04)	Secretory (0.945)
B2	CAHS1	26,979	1,167	237	38	6.4	No match	Cytoplasmic (0.932)
	CAHS2	24,587	918	216	53	6.1	No match	Cytoplasmic (0.948)
B3	CAHS3	33,128	1,154	303	76	6.7	No match	Mitochondrial (0.706)

[a]Molecular weight (MW) after removal of the signal peptide predicted by the SignalP program.

[b]In BLASTP search against non-redundant protein database, SAHS1 and SAHS2 showed weak similarity with *Pongo abelii* fatty acid binding protein (Pa_FABP, NP_001125017) and *Branchiostoma belcheri* FABP (Bb_FABP, ADD10136), respectively.

[c]Subcellular localizations were predicted by TargetP program.
